# Epidemiology & Infection goes open access

**DOI:** 10.1017/S0950268818003047

**Published:** 2018-11-28

**Authors:** Norman Noah, Fiona G. Hutton

**Affiliations:** 1 London School of Hygiene & Tropical Medicine, Keppel Street, London, UK; 2 Cambridge University Press, Cambridge, UK

The *Journal of Hygiene* was founded in 1901, and changed its name to *Epidemiology & Infection* in January 1987. The change occurred when the resident Editor, J.R. Pattison, was invited to the launch of a toothpaste at the Ritz Hotel in London [1]. He decided that using the word ‘hygiene’ was a distraction from the breadth of content that the journal published and changed the name to reflect a broad-based epidemiology and microbiology journal publishing research concerning the occurrence and spread of microbial diseases (hence ‘epidemiology *and* infection’) [1]. *Epidemiology & Infection* thrived under the changes, as global and national outbreaks, new microbes, techniques and methodology emerged.

Since the 1980s, scholarly publishing itself has continued to evolve; the most important changes occurred after the dawn of the internet, which has resulted in multiple exciting innovations and changes to the journal publishing ecosystem. One of these is the emergence of open access (OA) (see Peter Suber's comprehensive overview [2]), and the growth of OA journals; there are now over 12 000 fully OA, peer-reviewed scholarly journals in the world [3], constituting about a third of all peer-reviewed journals. These journals contain over 3.3 million OA articles [3].

It is with these changes in mind that we are thrilled to announce that *Epidemiology & Infection* will convert to the OA model of publication starting from 1st January 2019. From that date, *Epidemiology & Infection* will publish all articles under the Creative Commons Attribution License (CC-BY), which permits use, distribution, reproduction and adaptation in any medium, provided the original work is properly cited. The decision to flip *Epidemiology & Infection* from the subscription business model to the OA model was taken under the recognition that authors in this discipline are increasingly choosing to make their work available OA through a CC-BY license. A CC-BY license allows anyone anywhere in the world to read, use and cite the research, encouraging wider impact, collaboration and visibility. As a University Press, our priority is to focus on our authors and to provide the best quality journal to serve the community, regardless of the business model; however, it has become clear to us through recent analysis of papers published in *Epidemiology & Infection* that OA papers (published through the hybrid model) achieve greater downloads and become more highly cited. There is increasing evidence of this in other journals [4]. In tandem, an increasing number of our authors state that their funders want them to publish their output in gold OA, with some of those funders requiring their authors to publish only in fully gold OA journals. This is a trend we are seeing reflected across the scholarly communications landscape. Some institutes in Europe, and a few in the USA, have expressed support for the transformation of scholarly publishing from a subscription to an OA model. On 4th September 2018, a group of eleven European national research funders and the European Commission announced the formation of cOALition S to implement a bold new OA policy – Plan S [5], which includes 10 core principles. Together, these funders have made a commitment that by 2020, scientific publications that result from research funded by public grants provided by participating national and European research councils and funding bodies, must be published in compliant OA journals or on compliant OA platforms. Plan S also states that the hybrid model is no longer compliant [5].

Furthermore, there has been an increasing prevalence of ‘offsetting’ agreements. National consortia of libraries in Europe have been negotiating agreements with publishers that combine subscription and OA payments in a single deal, allowing their researchers to publish on a gold OA basis at no additional cost; everything is paid upfront as a lump sum. On 21 June 2018, the University of California's Systemwide Library and Scholarly Information Advisory Committee (SLASIAC) issued a Call to Action [6] ‘We believe the time has come to address these issues head-on through a combined strategy that places the need to reduce the University's expenditures for academic journal subscriptions in the service of the larger goal of transforming journal publishing to open access. Through our renewal negotiations with publishers, we will pursue this goal along two complementary paths: by reducing our subscription expenditures, and investing in open access support’. MIT, which has long been an important leader on OA in the USA, also recently signed a ‘Read and Publish’ agreement with the Royal Society for Chemistry (RSC) [7]. The aim is that, over time, as more universities adopt this type of contract, the proportion of paywalled articles will decline and funding will shift to supporting OA [7].

Cambridge University Press (CUP), as a not-for-profit publisher and a department of a major research-intensive university, occupies a unique niche in the publishing ecosystem. CUP publishes a range of journals in a variety of subject areas. Some of these titles have high OA uptake, reflecting the push towards publishing OA publication by certain communities; however, other journals have very low uptake of OA, reflecting the slowness and challenges found by their communities to support the OA model of publication. It is understood that the passage towards OA will take different directions in different disciplines, and will also depend on geographical location of authors.

At *Epidemiology & Infection* however we believe that publishing content OA is fundamentally important to our core mission of enabling the research that we publish be reused and disseminated widely. We know that we currently publish work that would be of substantial benefit to those not able to access the journal under the current subscription model, and so we are delighted to make the decision to unlock the potential of our high quality research papers, benefitting our discipline and enabling the journal to flourish. The core mission of CUP is to work towards a more impactful, community led and diverse open future. Flipping journals to OA in specific disciplines is a key part of that goal.

As a result of this change, an article processing charge will be payable by authors or their funder on acceptance of their primary research article. In the majority of cases, these costs are paid by the author, his or her institution, or a funder. *Epidemiology & Infection* will also be published under a continuous publication model, which means articles will be published as soon as they are ready, without the requirement to await composition in an issue, with issue numbers. This moves the journal away from the hangover of a print publication model to an online approach.

We hope you are as excited by this change as we are, as we continue to bring high quality and relevant content to our readers, providing a journal that is authoritative resource of global research in the occurrence and spread of microbial disease in humans and animals.

*Epidemiology & Infection* will continue to concentrate on its main themes of the epidemiology of infectious diseases in humans. It is one of the few journals which primarily straddle both these subjects of epidemiology and infection, and will continue to do so in the foreseeable future. In a world in which new infections and microbes seem to be reappearing, where new infections emerge and old ones resurge, and in which microbes become increasingly resistant to the agents we have to combat them, a journal such as this is needed even more than ever before.
